# Health risk assessment of ciprofloxacin, tetracycline, and oxytetracycline residues in raw, frozen, and boiled broiler chicken available in a local area of Bangladesh

**DOI:** 10.3389/frabi.2024.1364946

**Published:** 2024-04-03

**Authors:** Shaila Haque, Md. Yusuf Jamil, Md. Shahinul Haque Khan, Md. Sajib Al Reza, Md. Esrafil, Md. Zainul Abedin, Md. Abu Zubair, Md. Asaduzzaman Sikder, Luthfunnesa Bari

**Affiliations:** ^1^ Department of Food Technology and Nutritional Science, Mawlana Bhashani Science and Technology University, Tangail, Bangladesh; ^2^ Department of Chemistry, Bangladesh University of Health Science, Dhaka, Bangladesh; ^3^ Department of Biochemistry and Molecular Biology, Mawlana Bhashani Science and Technology University, Tangail, Bangladesh

**Keywords:** health risk, broiler chicken, ciprofloxacin, tetracycline, oxytetracycline, HPLC, hazard index

## Abstract

**Introduction:**

The misuse of antibiotics in poultry farming is a global issue.

**Objective:**

The focus of this study was the health risk assessment of consumers from the determination of ciprofloxacin (CIP), tetracycline (TC), and oxytetracycline (OTC) in broiler chicken in the raw, frozen, and boiled stages using solid-phase extraction, high-performance liquid chromatography, and ultraviolet detection (SPE-HPLC-UV).

**Materials and methods:**

Chromatographic separation was achieved using 0.3% metaphosphoric acid and acetonitrile (1:10, *v*/*v*) for CIP at 280 nm and oxalic acid (0.01 M) and acetonitrile (1:1, *v*/*v*) for TC and OTC at 355 nm with different retention times. The method had an acceptable precision with good linearity, specificity, limit of detection, limit of quantification, accuracy, and stability.

**Results:**

Among a total of 252 raw samples, approximately 68.25%, 25.4%, and 7.54% contained CIP, TC, and OTC, respectively. Out of the positive raw samples, CIP exceeded the maximum residual limit (MRL) in 3.6% muscle, 14.3% liver and 17.9% skin samples, whereas TC and OTC were below the MRLs. The residual concentrations of these antibiotics were almost unchanged in frozen samples. After boiling the chicken samples, the TC and OTC residues were reduced significantly compared to CIP. Although the concentrations of CIP in boiled samples were above the MRL set by the European Union, these did not exceed the hazard index 1.

**Conclusion:**

Based on these results, the exposure levels to antibiotics in broiler chicken meats may be considered to have a low risk for human health.

## Introduction

Since their discovery and development, antibiotics have been frequently used as veterinary and human medicines to treat various infectious diseases (Samanidou et al., 2007). They can either have activity against a broad spectrum of microorganisms or can be active against a small extent of specific organisms (Samanidou et al., 2006; Samanidou and Evaggelopoulou, 2015). As veterinary drugs, antibiotics play a vital role in the maximal production of industrial farm animals and in reducing the morbidity and mortality rates among livestock (Page and Gautier, 2012). Ciprofloxacin (CIP), tetracycline (TC), and oxytetracycline (OTC) are the most frequently applied veterinary drugs in Bangladesh. The main causes of antibiotic residues include the unauthorized use and unrestricted administration of veterinary medications to healthy animals, as well as failing to consider the withdrawal period, not seeking professional advice, using medications beyond the recommended dosage, administering medications frequently, and increasing dosing (Baran et al., 2011; Croubels and Daeseleire, 2012; Bitas et al., 2018). These residues have the potential to be hazardous to human health and can trigger allergic reactions. In addition, the prolonged accumulation of CIP, TC, and OTC residues can lead to the growth of microorganisms that cause antibiotic resistance (Garcia et al., 2004; Fan et al., 2020). To ensure the reduction of the quantity of veterinary drugs from the edible parts of farm animals, a minimum withdrawal time between the administration of antibiotics and the slaughter has been established (Croubels and Daeseleire, 2012). The European Union (EU) has established maximum residual limits (MRLs) for residues of veterinary pharmaceuticals, including CIP, TC, and OTC, in animal tissues and derivative foods entering the human food chain in Council Regulation EEC 37/2010 and its later modification [Commission Regulation (EU) No. 37/2010 of 22 December 2009, L.15/1-72]. Monitoring is therefore required to ensure that antibacterial agents are not present in quantities that could endanger the health of the general public. Recently, multi-residue techniques have been created for a variety of residue classes observed in animal tissues, including fluoroquinolones (Schneider et al., 2005; Verdon et al., 2005) and TCs (Anderson et al., 2003; Cinquina et al., 2003a). HPLC has a reputation for being a reliable and highly selective separative tool (Almalki et al., 2021) for detecting antibiotic residues. Although a lot of initiatives have been taken from government regulatory bodies, the misuse of antibiotics is still occurring. The presence of antibiotic residues in poultry food has become a matter of concern as its resistance is considered as a health risk. Therefore, annual monitoring is mandatory. The aims of the present study were to determine CIP, TC, and OTC residues from poultry samples collected from Tangail Sadar Upazila using HPLC and to assess their risks to consumer health.

## Experimental

### Sampling

For this study, a total of 252 breast muscle, liver, and skin samples at raw, cooked, and frozen stages were prepared from 56 fresh broiler chickens. These chickens, weighing 1.0–2.0 kg, were purchased from seven traditional local bazaars in Tangail Sadar Upazila, Bangladesh. Upon collection, all samples were first defeathered and placed in clean, sterile polyethylene bags, which were then transported to the laboratory as quickly as possible. Frozen samples were then immediately stored in a refrigerator (WFA-2A3-ELXX-XX, Walton, Bangladesh) at −2°C for 3 days to determine the quantity of antibiotic residues in each sample.

### Chemicals and reagents

Ciprofloxacin hydrochloride (C_17_H_21_CLFN_3_O_4_, 385.81 g/mol), a fine crystalline powder, and 98% oxytetracycline hydrochloride (C_22_H_24_N_2_O_9_·HCL, 496.90 g/mol), a yellow crystalline powder, were purchased from the International Laboratory (South San Francisco, CA, USA). Tetracycline hydrochloride (C_22_H_24_N_2_O_8_·HCL, 480.91 g/mol) was obtained from Alfa Aesar (Thermo Fisher Scientific, Lancashire, UK). HPLC grade methanol was purchased from Thermo Scientific™ (Princeton, NJ, USA), and acetonitrile was manufactured by J.T.Baker^®^, Avantor^®^ Performance Materials LLC (Center Valley, PA, USA). Analytical grade oxalic acid (HO_2_CCO_2_H·2H_2_O, 126.07 g/mol) was from BDH Laboratory Supplies (Poole, UK). HPLC grade acetic acid (CH_3_COOH, 100%) was purchased from Sigma-Aldrich (Merck, Darmstadt, Germany). Meta-phosphoric acid (HPO_3_, 39%–43%) was obtained from Alfa Aesar (Thermo Fisher Scientific, Lancashire, UK). Distilled and deionized water, with resistivity >18 MΩ cm^−1^ at 25°C and total organic carbon (TOC) <5 ppb before extraction in the laboratory, was obtained from Labconco 9000620 WaterPro PS-series (Kansas City, MO, USA).

### Instrumentation

For the chromatographic determination of CIP, TC, and OTC, an LC-20AT pump equipped with a Shimadzu CBM-20A system controller (Kyoto, Japan) permitting an automated operation that delivers the solvent system to the analytical column was employed. A Rok 5 µm C18, 100 Å, 250 × 4.6-mm analytical column (Restek, PA, USA) was used. Injection of the sample was manually accomplished through a Rheodyne™ 7125i injection valve equipped with a 20-µl loop. Detection was performed using a UV detector at a wavelength of 280 nm for CIP and at 355 nm for TC and OTC, which complied with the LabSolutions software by Shimadzu for data acquisition. Mobile phases were degassed with a glass vacuum filter (IDEX Corporation, Benton Harbor, MI, USA) using Whatman filter paper (PALL Corporation, Mumbai, India). A TRXA-C vortex (TRIU, China) and a UBT-580 ultrasonic bath (Unilab, USA) were used for proper homogenization. The samples were extracted with a 50-ml Sorvall-ST-8R centrifuge (Thermo Fisher Scientific, Osterode, Germany) and a rotary vacuum evaporator (2610000; Witeg, Wertheim, Germany). A 3-ml Resprep C18 cartridge (26031; Restek, PA, USA) was used for the cleanup procedure. **Table 1** shows the experimental conditions for HPLC for the determination of CIP, TC, and OTC.

**Table 1:** Chromatographic conditions for HPLC for the determination of ciprofloxacin (CIP), tetracycline (TC), and oxytetracycline (OTC).

HPLC system	Conditions		
	CIP	TC	OTC
Column	Restek C18 (250 × 4.6, 5 µm)	Restek C18 (250 × 4.6, 5 µm)	Restek C18 (250 × 4.6, 5 µm)
Solvent system	2% acetic acid and 95% acetonitrile	Methanol, acetonitrile, and oxalic acid (0.01 M)	Methanol, acetonitrile, and oxalic acid (0.01 M)
Solvent system ratio	83:17, *v*/*v*	5:18:77, *v*/*v*/*v*	5:18:77, *v*/*v*/*v*
Flow rate (ml/min)	1	1	1
Injection volume (µl)	20	20	20
Pump pressure (kg/m^2^)	130–137	128–134	128–134
Wavelength	280 nm	355 nm	355 nm
Retention time	8.6 ± 0.4	8.7 ± 0.4	6.7 ± 0.4
Run time (min)	12	12	12
Temperature	Ambient	Ambient	Ambient

### Preparation of standard and sample extraction

Aqueous stock solutions of 100 µg/ml were prepared by taking 5 mg of each CIP, TC, and OTC standard in a 50-ml volumetric flux and made up to the mark by dissolving them (2% acetic acid and 100% acetonitrile in a ratio 82:18, *v*/*v*, for CIP and pure methanol for TC and OTC). The stock solutions were found to be stable for at least 1 month when wrapped with paraffin wax and stored at refrigerated temperature (4°C). Working standard concentrations of 0.0195, 0.039, 0.078, 0.156, 0.195, 0.3125, 0.39, 0.625, 0.78, 1.25, 1.56, 2.5, 3.125, and 5 µg/ml were prepared from stock solution using the same dilution solvents.

A precise weight of 5 ± 0.02 g by analytical balance (PH224; OHAUS Corporation, Parsippany, NJ, USA) from each muscle, liver, and skin sample at three stages (i.e., raw, boiled, and frozen) was blended separately using a blender (MX-AC300; Panasonic Corporation, Gurugram, India) or smashed with a mortar and pestle and then transferred into a 50-ml centrifuge tube. Fortified samples were prepared by adding the working solutions of CIP, TC, and OTC at various concentrations with the smashed samples. Subsequently, 15 ml of the extraction solvent [0.3% meta-phosphoric acid and acetonitrile (1:10, *v*/*v*) for CIP and 0.01 M oxalic acid and acetonitrile (1:1, *v*/*v*) for TC and OTC] was added into the centrifuge tube. The mixture was homogenized using a vortex for approximately 10 min, followed by sonication at 30°C for 10 min. The mixture was then centrifuged at 9,000 rpm at 21°C for approximately 10 min. The residual liquid was centrifuged again under the same conditions. For CIP determination, the supernatant was collected with a 1,000-µl micropipette from the rotary evaporator flask, which was partially evaporated at 40°C for 3–4 min prior to centrifugation. Solid phase extraction (SPE) was carried out with the help of C18 cartridges for the cleanup procedure, which was preconditioned by passing 3 ml methanol and 3 ml HPLC grade deionized water. The sample was then filtered using a Sterlitech (Kent, WA, USA), 25 mm, 0.22 µm, hydrophilic (PTFE) syringe filter, and an aliquot of 20 µl was injected into the HPLC system. The frozen (−2°C) and boiled (100°C) samples were prepared using the same process after thawing and cooling at room temperature.

### Method validation

The proposed HPLC analytical method was appropriately evaluated in terms of specificity, linearity, sensitivity, accuracy, precision, and stability using both working standard solutions and spiked samples of muscle, liver, and skin in accordance with the European Community’s defined validation protocol for antibiotic residues in food items of animal origin (Communities, 2002).

Linearity evaluation was performed by injecting seven working standards (range, 0.0195–5 µg/ml) for CIP and five different concentrations each for TC and OTC (0.195, 0.39, 0.78, 1.56, and 3.125 µg/ml) with two replicate injections. The working concentrations were prepared by diluting the stock solutions with dilution solvents. The calibration curves with respective correlation coefficients (*R* ^2^) were obtained by linear regression analysis. In terms of recovery, accuracy analysis was performed in spiked samples with working standards at four concentration levels (i.e., 250, 500, 1,250, and 2,000 µg/kg) for CIP and at three concentration levels (i.e., 500, 1,000, and 1,250 µg/kg) each for TC and OTC. Repeatability for precision study was performed by diluting the stock standard solution at different concentrations. The intra-assay repeatability at two concentration levels was analyzed three different times within a day. Inter-assay repeatability was performed comparing three consecutive days by assessing the same concentrations. The results were expressed using relative standard deviation. The limit of detection (LOD) and limit of quantification (LOQ) were used to assess the sensitivity of the method. The LOD and LOQ were established at concentrations where, under the circumstances of our HPLC, the signal-to-noise (S/N) ratios were equal to 3.3 and 10, respectively, for each antibiotic residue in broiler chicken samples (i.e., muscle, liver, and skin). The LOD and LOQ were calculated based on S/N ratios occupying the following equations, where *σ* is the SD of the response and *s* is the slope of the calibration curve. Specificity of the analytical approach can distinguish between the analytes of interest and other compounds that are likely to be present in the sample matrix. The interference of other compounds present in the matrix was verified by injecting 15 different samples for each antibiotic, including a blank, standard, and a spiked sample (five for each). In the case of the HPLC procedure, full separation of the analyte peaks from other interfering signals originated in the sample matrix was ascertained. Since the stability of stock and matrix constituents during analysis at storage may cause significant deviations in the results, monitoring the stability is of paramount importance. For this, both the standard and the sample matrix were verified at room temperature at 0 h and after 24 h at 25°C and 4°C. Three replicate injections were performed each time.

### Health risk assessments

Daily exposure to CIP, TC, and OTC of the human body from edible parts of poultry chicken was assessed by calculating the estimated daily intake (EDI), as described by Moudgil et al. (2019a): EDI (µg/kg body weight per day) = *C* × DIF/BW, where *C* is the mean concentration of the antibiotic in poultry (in micrograms per gram), DIF is the daily intake of food (in grams per day), and BW is the mean body weight (bw) of adult (60 kg). To calculate EDI, the maximum mean concentration of the antibiotic was considered. According to FAO/WHO, the average daily intake (ADI) for CIP is 2 µg/kg bw per day and that for both TC and OTC is 30 µg/kg bw per day. The Report of the Household Income and Expenditure Survey (2016) published that the poultry meat consumption rate for a 60-kg Bangladeshi adult is 17.33 g/day (Ahmed et al., 2022). In Bangladesh, the consumption rate of liver tissue was estimated at 3 g/person per day (Zeinali et al., 2019). To predict the long-term effects on health, a hazard index (HI) was calculated using the equation HI = EDI/ADI. HI less than 1 denotes that the poultry meat is safe for human consumption. However, 1 ≤ HI ≤ 10 warns a risk, but not emergency, while HI > 10 indicates an unacceptable risk for consumption (Oyedeji et al., 2019).

### Statistical analysis

For this research, a descriptive analysis encompassing the mean, standard deviation (SD), and relative standard deviation (RSD) was performed and analyzed using Microsoft Excel 2010. A *t*-test was conducted using SPSS version 25.

## Results

### Validation and method performance

**Figure 1:**
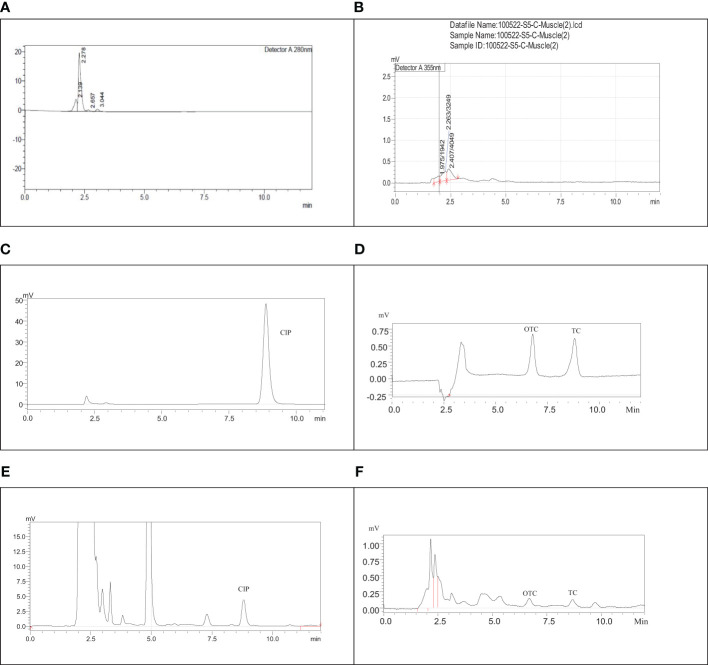
Chromatograms of the blank ciprofloxacin (CIP) **(A)**, blank tetracycline (TC) and oxytetracycline (OTC) **(B)**, CIP standard **(C)**, TC and OTC standard **(D)**, muscle sample spiked with CIP **(E)**, and the skin sample spiked with TC and OTC **(F)**.

**Figure 2:**
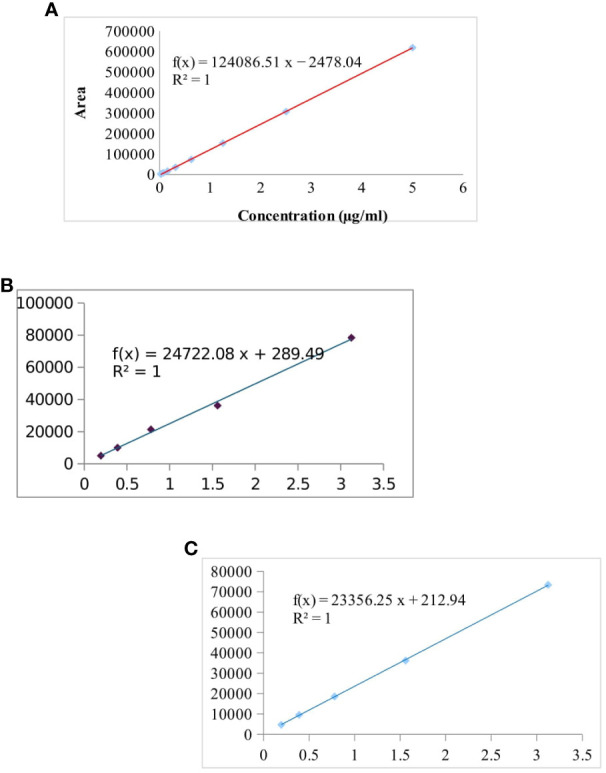
Calibration curve of the ciprofloxacin (CIP) standard (from 0.0195 to 5 µg/ml) **(A)**, tetracycline (TC) standard **(B)**, and oxytetracycline (OTC) standard (0.195 to 3.125 µg/ml for both TC and OTC) **(C)**.

The separation and specificity of the SPE-HPLC-UV method for the analysis of CIP, TC, and OTC residues are illustrated in **Figure 1** by comparing the chromatograms of the blank, standard, and the sample spiked with antibiotics. No endogenous compound present in the sample matrix appeared to interfere at the retention times of the CIP (8.5 ± 0.4, 280 nm), TC (8.9 ± 0.3, 355 nm), and OTC (6.9 ± 0.3, 355 nm) peaks, showing that the method was extremely specific for this analysis. The chicken samples containing antibiotic residues were spiked with each standard. The obtained peak area was increased to a significant level, which indicated the specificity of the method. The calibration curves in the concentration range from 0.0195 to 5 µg/ml for CIP and from 0.195 to 3.125 µg/ml for both TC and OTC standards showed good linearity (**Figure 2**).

**Table 2** shows the highly significant correlation coefficient (*R* ^2^) values ranging from 0.9964 to 0.9999 from the linear regression for each antibiotic. The accuracy of the CIP-spiked samples was between 60.53% and 77.37%, while that for the TC- and OTC-spiked samples was 85.26%–99.87%. The RSD value of each spiked sample was less than 2, which showed good accuracy of the methods, in accordance with the acceptance criteria set out in the European Commission Decision 2002/657 (Communities, 2002). The LOD and LOQ were found to be 3.8–20.8 and 12.2–69.5 µg/kg, respectively, for the three selected antibiotics (**Table 2**).

**Table 2:** Analytical features of the method.

Antibiotic	Sample (µg/kg)	Accuracy			LOD (µg/kg)	LOQ (µg/kg)	*R* ^2^	Linearity
		Found conc. ± SD	Mean recovery (%)	RSD (%)				
CIP	Muscle							
	250	168.18 ± 1.28	67.27	0.76				
	500	336.43 ± 1.79	67.29	0.53	9.715	29.439	0.9999	113,690*x* − 4,358.3
	1,250	813.2 ± 1.81	65.06	0.22				
	Liver							
	250	162.09 ± 2.67	64.84	1.65				
	500	325.38 ± 2.16	65.08	0.66	18.949	57.421	0.9993	103,198*x* + 2,005.5
	1,250	796.02 ± 8.71	63.68	1.09				
	Skin							
	250	193.42 ± 3.01	77.37	1.55				
	500	336.97 ± 4.47	67.39	1.33	10.398	31.503	0.9966	126,528*x* − 3,525
	1,250	756.67 ± 12.39	60.53	1.64				
TC	Muscle							
	500	459.62 ± 3.6	91.92	0.78				
	1,000	852.60 ± 7.6	85.26	0.89	10.8	35.9	0.9964	6.525*x* + 521.24
	1,250	1,100.39 ± 14.2	88.03	1.29				
	Liver							
	500	496.31 ± 1.0	99.26	0.20				
	1,000	927.89 ± 11.4	92.79	1.23	3.8	12.2	0.9997	6.5301*x* + 877.2
	1,250	1,128.26 ± 15.4	90.26	1.36				
	Skin							
	500	492.81 ± 6.7	98.56	1.36				
	1,000	934.75 ± 12.9	93.47	1.38	20.8	69.5	0.9999	6.7373*x* + 740.7
	1,250	1,145.65 ± 7.2	91.65	0.63				
OTC	Muscle							
	500	449.95 ± 4.9	89.99	1.09				
	1,000	907.65 ± 6.6	90.77	0.72	15.2	50.7	0.996	7.1183*x* − 115.8
	1,250	1,215.10 ± 13.1	97.21	1.08				
	Liver							
	500	431.24 ± 4.2	86.25	0.98				
	1,000	908.84 ± 13.9	90.88	1.53	12.2	40.8	0.9951	6.4459*x* + 204.1
	1,250	1,084.81 ± 4.6	86.78	0.43				
	Skin							
	500	475.06 ± 1.1	95.01	0.23				
	1,000	998.72 ± 10.9	99.87	1.09	4.0	13.3	0.9963	7.1373*x* + 176
	1,250	1,200.03 ± 9.1	96.00	0.76				

RSD, relative standard deviation; LOD, limit of detection; LOQ, limit of quantification.

The RSDs of the intra- and inter-day precision of the spiked chicken samples were 1.10%–4.57% and 1.18%–4.64% for CIP, 1%–4.09% and 1.31%–5.74% for TC, and 0.30%–1.28% and 1.29%–3.28% for OTC, respectively, as presented in **Table 3**. The recovery of CIP observed 62.50%–70.41% in intra-day and 64.58%–70.58% in inter-day precision (**Table 3**). For TC and OTC, the recoveries (92.52%–101.22%) from the skin were higher than those from the muscle and liver (79.27%–96.70%). These results on precision met the criteria set out in the European Commission Decision 2002/657 (Communities, 2002). The stability studies of the methods for the CIP-, TC-, and OTC-spiked samples are summarized in **Table 4**. At different times and temperatures, the mean recovery and the %RSD of CIP, TC, and OTC in the muscle were 65.27%–92.12% and 0.52%–2.83%, respectively. In the liver, the mean recovery was 60.92%–93.90%, whereas this was higher in the skin, which ranged 67.17%–106.2%; the RSD was below 2% in both samples of CIP, TC, and OTC.

**Table 3:** Intra- and inter-day precision of spiked muscle, liver, and skin samples.

		Added conc. (µg/kg)	Intra-day (*n* = 9)			Inter-day (*n* = 9)		
			Found conc. ± SD	RSD (%)	Recovery (%)	Found conc. ± SD	RSD (%)	Recovery (%)
CIP	Muscle	500	326.89 ± 9.346	2.86	65.30	333.16 ± 13.73	3.73	66.94
		1,250	824.86 ± 9.099	1.10	65.99	846.31 ± 18.43	2.18	67.70
	Liver	500	325.76 ± 4.11	1.26	65.15	337.03 ± 15.96	4.64	67.41
		1,250	781.22 ± 34.30	4.39	62.50	807.34 ± 35.68	4.37	64.58
	Skin	500	352.03 ± 16.09	4.57	70.41	351.97 ± 20.28	4.49	70.58
		1,250	846.004 ± 27.8	3.29	67.68	847.31 ± 17.717	1.18	67.79
TC	Muscle	1,000	792.74 ± 8.5	4.09	79.27	815.30 ± 9.9	5.24	81.53
		1,250	1,108.0 ± 13.3	2.38	88.64	1,115.83 ± 10.7	2.84	89.27
	Liver	1,000	885.47 ± 12.1	1.96	88.55	921.31 ± 12.1	1.31	92.13
		1,250	1,067.06 ± 35.1	3.29	85.37	1,123.0 ± 39.0	3.47	89.84
	Skin	1,000	935.98 ± 10.7	1.14	93.60	1,026.66 ± 59.0	5.74	102.67
		1,250	1,156.55 ± 11.6	1.00	92.52	1,181.50 ± 33.0	2.80	94.52
OTC	Muscle	1,000	892.61 ± 4.48	0.50	89.26	915.72 ± 14.5	2.31	92.16
		1,250	1,208.71 ± 10.63	0.88	96.70	1,252.75 ± 25.3	2.02	100.22
	Liver	1,000	923.16 ± 11.8	1.28	92.32	952.27 ± 12.3	1.29	95.23
		1,250	1,101.48 ± 12.1	1.10	88.12	1,166.77 ± 38.3	3.28	93.34
	Skin	1,000	1,012.18 ± 8.38	0.83	101.22	1,030.37 ± 14.6	1.42	103.04
		1,250	1,187.35 ± 3.58	0.30	94.99	1,222.85 ± 29.51	2.41	97.83

RSD, relative standard deviation; CIP, ciprofloxacin; TC, tetracycline; OTC, oxytetracycline.

**Table 4:** Stability of the examined ciprofloxacin (CIP), tetracycline (TC), and oxytetracycline (OTC) in poultry muscle, liver, and skin after 24 h at room temperature and at refrigerated temperature.

	Injection interval	Added conc. (µg/kg)	No. of replicates	Muscle		Liver		Skin	
				RSD (%)	Mean recovery (%)	RSD (%)	Mean recovery (%)	RSD (%)	Mean recovery (%)
CIP	0 h	1,250	3	0.52	68.96	1.83	70.28	0.91	68.24
	After 24 h at 25°C	1,250	3	2.83	67.12	0.92	68.48	1.64	67.17
	After 24 h at 4°C	1,250	3	0.79	65.27	1.77	60.92	1.88	68.01
TC	0 h	1,000	3	1.66	81.07	1.16	90.44	0.86	106.2
	After 24 h at 25°C	1,000	3	1.59	79.84	1.92	92.33	0.04	102.0
	After 24 h at 4°C	1,000	3	1.97	80.46	1.92	89.98	1.77	98.75
OTC	0 h	1,000	3	1.04	92.12	0.30	93.90	0.97	104.8
	After 24 h at 25°C	1,000	3	0.57	88.63	1.30	90.82	1.38	100.2
	After 24 h at 4°C	1,000	3	0.98	88.23	1.79	90.90	1.57	102.4

RSD, relative standard deviation.

### CIP, TC, and OTC residues

**Figure 3:**
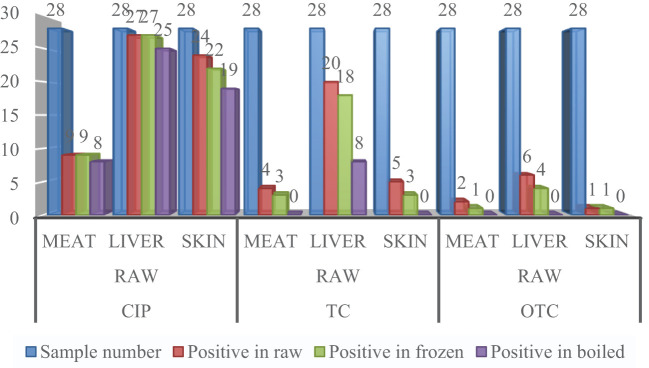
Frequency of ciprofloxacin (CIP), tetracycline (TC), and oxytetracycline (OTC) residues in a number of chicken meat, liver, and skin samples.

**Figure 4:**
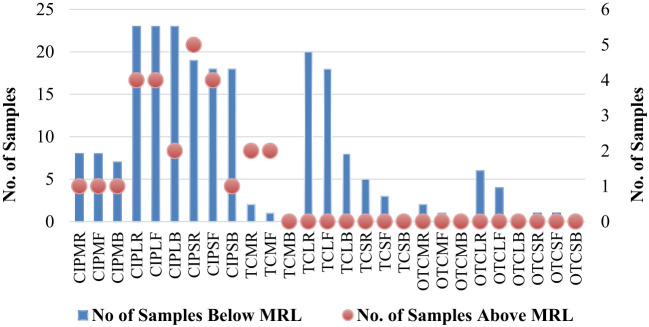
Comparison between below and above MRL of the number of positive antibiotic residues. *CIPMR*, ciprofloxacin-muscle-raw; *CIPMF*, ciprofloxacin-muscle-frozen; *CIPMB*, ciprofloxacin-muscle-boiled; *CIPLR*, ciprofloxacin-liver-raw; *CIPLF*, ciprofloxacin-liver-frozen; *CIPLB*, ciprofloxacin-liver-boiled; *CIPSR*, ciprofloxacin-skin-raw; *CIPSF*, ciprofloxacin-skin-frozen; *CIPSB*, ciprofloxacin-skin-boiled; *TCMR*, tetracycline-muscle-raw; *TCMF*, tetracycline-muscle-frozen; *TCMB*, tetracycline-muscle-boiled; *TCLR*, tetracycline-liver-raw; *TCLF*, tetracycline-liver-frozen; *TCLB*, tetracycline-liver-boiled; *TCSR*, tetracycline-skin-raw; *TCSF*, tetracycline-skin-frozen; *TCSB*, tetracycline-skin-boiled; *OTCMR*, oxytetracycline-muscle-raw; *OTCMF*, oxytetracycline-muscle-frozen; *OTCMB*, oxytetracycline-muscle-boiled; *OTCLR*, oxytetracycline-liver-raw; *OTCLF*, oxytetracycline-liver-frozen; *OTCLB*, oxytetracycline-liver-boiled; *OTCSR*, oxytetracycline-skin-raw; *OTCSF*, oxytetracycline-skin-frozen; *OTCSB*, oxytetracycline-skin-boiled.

A total of 504 samples of muscle, liver, and skin in three different stages from 56 broiler chickens (28 for CIP and 28 for TC and OTC) were examined using the validated HPLC method for the determination of CIP, TC, and OTC. **Figure 1** shows the chromatograms of CIP, TC, and OTC of the standard and chicken samples. **Figure 3** presents the frequency of CIP, TC, and OTC residues in chicken meat, liver, and skin samples at various stages. The analytical results of the CIP, TC, and OTC residues in the muscle, liver, and skin of chicken at three different stages are shown in **Table 5** according to the permissible MRLs set by EU Council Regulation no. 2377/90. CIP residues were found in 22.79–341.8 µg/kg in the muscle, 22.83–2,087.296 µg/kg in the liver, and 15.33–484.2 µg/kg in the skin. The assessment of CIP in chicken samples showed that 96.43% of raw liver contained the highest CIP residues, followed by raw skin (85.71%) and muscle (32.14%). Among the positive CIP samples in the raw stage, 14.3% liver, 17.9% skin, and 3.6% muscle exceeded the MRLs. In the frozen stage, no significant change of the CIP residues was found in the muscle and the liver, except for the skin where the CIP residues were reduced moderately after boiling in the liver (7.1%) and the skin (3.6%). Although the CIP residue in muscle was decreased significantly after boiling, it remained above the MRL. **Figure 4** shows the comparison between below and above MRL of the number of positive CIP, TC, and OTC residues.

**Table 5:** Analytical results of the ciprofloxacin (CIP), tetracycline (TC), and oxytetracycline (OTC) residues in raw, frozen, and boiled chicken samples.

		Sample	Sample size	Positive samples (%)	Min (µg/kg)	Max (µg/kg)	Mean conc. (µg/kg)	MRL (µg/kg)	Below MRL (%)	Above MRL (%)
CIP	Muscle	Raw	28	32.14	28.52	341.8	74.49b		28.57	3.6
		Frozen	28	32.14	22.92	339.2	67.59b		28.57	3.6
		Boiled	28	28.57	22.79	107.7	50.72a	100	25.00	3.6
	Liver	Raw	28	96.43	28.32	2,087.3	184.6b		82.11	14.3
		Frozen	28	96.43	28.19	1,951.4	163.6ab		82.11	14.3
		Boiled	28	89.29	22.83	982.3	140.4a	200	82.11	7.1
	Skin	Raw	28	85.71	25.25	484.2	79.75c		67.83	17.9
		Frozen	28	78.57	15.33	253.2	68.47b		64.28	14.3
		Boiled	28	67.86	15.76	117.1	49.15a	100	64.28	3.6
TC	Muscle	Raw	28	14.3	58.3	353.1	180.0a		7.1	7.1
		Frozen	28	10.71	12.5	341.5	171.4a	200	3.6	7.1
		Boiled	28	0	0	0	0		0	0
	Liver	Raw	28	71.4	64.1	504.1	172.2c		71.4	–
		Frozen	28	64.28	16.9	463.0	134.2b	600	64.28	–
		Boiled	28	28.57	37.5	126.0	34.3a		28.57	–
	Skin	Raw	28	17.9	34.3	249.8	98.1b		17.9	–
		Frozen	28	10.71	15.4	105.2	35.6a	200	10.71	–
		Boiled	28	0	0	0	0		0	–
OTC	Muscle	Raw	28	7.1	40	65	52b		7.1	–
		Frozen	28	3.6	17.1	40.8	28.9a	200	3.6	–
		Boiled	28	0	0	0	0		0	–
	Liver	Raw	28	21.4	69.4	109.5	81.97b		21.4	–
		Frozen	28	14.3	20.4	54.1	33.9a	600	14.3	–
		Boiled	28	0	0	0	0		0	–
	Skin	Raw	28	3.6	49.4	49.4	49.4b		3.6	–
		Frozen	28	3.6	30	30	30a	200	3.6	–
		Boiled	28	0	0	0	0		0	–

Values in the same column with lowercase letters are significantly different (p < 0.05) for the different stages of the sample.

MRL, maximum residual limit.

**Table 5** presents the range of TC residues in the muscle, liver, and skin, which were 12.5–371.5, 16.9–504.9, and 15.4–249.8 µg/kg, respectively. The highest percentage of TC was observed in the liver (71.4%), followed by the skin (17.9%) and muscle (14.3%), in the raw condition. Among these, 7.1% of raw muscle contained TC residues above the MRL. In the frozen stage, the TC residues were reduced significantly in the liver and skin, except for the muscle sample (7.1%) showing above the MRL. After boiling, TC was remarkably reduced in the liver sample, without any residues detected in muscle and skin samples. Assessment of the OTC residues found 17.1–65 µg/kg in the muscle, 20.4–109.5 µg/kg in the liver, and 30–49.4 µg/kg in the skin. The highest percentage of OTC residues was found in raw liver, with a range of positive samples from 3.6% to 21.4%. It was noted that the values of all the OTC-positive samples were below the MRL. A significant reduction was found in OTC residues in the muscle, liver, and skin samples in the frozen stage, but no OTC residues were present in the samples after boiling.

### Health risk assessment

Health risk assessment of these three antibiotics was calculated from the ratio of the EDI of the CIP, TC, and OTC residues obtained from chicken (meat and liver) to ADI, which is, as well as the hazard index (HI), is presented in **Table 6**. The daily intake of chicken skin for a Bangladeshi individual and the ADI for antibiotics in the skin were unavailable. The HI investigated three antibiotic residues in broiler chicken at various stages, which was calculated by considering the maximum mean concentration of the CIP, TC, and OTC residues. **Table 6** shows that the EDI range of CIP in chicken meat and liver was 0.0311–0.1044 µg/kg bw per day, while the corresponding HI values ranged from 0.0156 to 0.0522. For TC, the EDI range was 0.0063–0.1020 µg/kg bw per day, while the HI ranged from 0.0 to 0.0034. The EDI and HI values for OTC ranged 0.0–0.0188 µg/kg bw per day and 0.0–0.0006, respectively. The highest EDI of CIP was found in the raw liver chicken sample, whereas the EDI value was found to be 0.00 µg/kg bw per day in boiled muscle for TC and in both boiled muscle and liver for OTC. These findings indicated that the EDI value calculated based on a dietary consumption containing the maximum mean concentrations of CIP, TC, and OTC was considerably lower than the ADI values of the antibiotic residues. The present experimental results showed that all HI values were below 0.0522.

**Table 6:** Health risks associated with ciprofloxacin (CIP), tetracycline (TC), and oxytetracycline (OTC) residues in edible parts of chicken at three different stages.

Antibiotic	Sample	Stage	EDI (µg/kg bw per day)	ADI (µg/kg bw per day)	HI (EDI/ADI)
CIP	Muscle	Raw	0.0987	2	0.0493
		Frozen	0.0980		0.0490
		Boiled	0.0311		0.0156
	Liver	Raw	0.1044	2	0.0522
		Frozen	0.0976		0.0488
		Boiled	0.0491		0.0246
	Skin	Raw	ND	NA	ND
		Frozen	ND		ND
		Boiled	ND		ND
TC	Muscle	Raw	0.1020	30	0.0034
		Frozen	0.0986		0.0033
		Boiled	0.0		0.0
	Liver	Raw	0.0252	30	0.0008
		Frozen	0.0232		0.0008
		Boiled	0.0063		0.0002
	Skin	Raw	ND	NA	ND
		Frozen	ND		ND
		Boiled	ND		ND
OTC	Muscle	Raw	0.0188	30	0.0006
		Frozen	0.0118		0.0004
		Boiled	0.0		0.0
	Liver	Raw	0.0055	30	0.0002
		Frozen	0.0027		0.0001
		Boiled	0.0		0.0
	Skin	Raw	ND	NA	ND
		Frozen	ND		ND
		Boiled	ND		ND

EDI, estimated daily intake; ADI, average daily intake; HI, hazard index; bw, body weight; ND, not detected; NA, not available.

## Discussion

The present study used SPE in sample preparation, and this required conditioning in order to moisten the sorbent and permit the liquid to penetrate the pores before the sample is introduced (Lu et al., 2019). In our approach, the wavelength used for CIP was consistent with Yorke and Froc (2000). For TC and OTC, a silica-based C_18_ analytical column, UV detection at 355 nm, flow rate of 1.0 ml/min, SPE C_18_ cartridges, and a 0.22-μm syringe filter were used following De Ruyck et al. (1999), Miranda et al. (2009); Shalaby et al. (2011), and Zhao et al. (2010). Christodoulou et al., (2007) found 53.5%–83.7% CIP recovery in egg yolk using different extraction solvents. A higher recovery of CIP was obtained in chicken muscle and liver by changing the matrix, extraction solvent, and their ratio (Posyniak et al., 2001). The recovery of OTC reported by Olatoye and Kayode (2012) was 80.5%–87.8%, which was somewhat lower, while Guo et al. (2012) reported 80.9%–119.5% recovery for both TC and OTC, which was higher than our findings. Strong acids and precipitating agents are recommended for deproteinization to improve the extraction of antibiotics from complex matrices as TCs readily bind to proteins (Pérez-Rodríguez et al., 2018). Our recovery values for the edible parts of chicken were comparable to those obtained by Blasco et al. (2009); Yu et al. (2011), and Masawat and Slater (2007). The LOD and LOQ values for TC and OTC were comparable to those obtained by Cornejo et al. (2017), Ozdemir Olgun and Demirata (2020), and Shalaby et al. (2011), but were higher than that in the study by Guo et al. (2012). According to Schneider et al. (2005), the LOD and LOQ of CIP were 1 and 5 µg/kg, respectively, which were smaller than those in our study. The findings for the precision values had similarities to others. Shalaby (1996) revealed a higher recovery in chicken muscle (98.4%–100.3%) and egg yolk (99.3%–101.6%) and lower RSD values (1.9%–3.1%) in chicken muscle and egg yolk (0.4%–2.5%) for intra-day precision. Schneider et al. (2007) reported below 4% RSD and 75%–90% recovery from muscle tissue in his findings. Another study stated that the recovery of intra-day precision for CIP in milk sample was 85.4%–89.1% and that the RSD was below 6% (Christodoulou et al., 2007). Yorke and Froc (2000) disclosed a 67% recovery and an 8.4% RSD as inter-day precision in chicken muscle, whereas Cinquina et al. (2003b) obtained 4.1% RSD. Yu et al. (2011) reported within- and between-day precision values for chicken samples of 6.2%–8.2%, 5.9%–8.1%, and 8.1%–9.8%, and 8.7%–8.8% for both TC and OTC, respectively. Blasco et al. (2009) reported higher RSD values for intra-day (i.e., 14.3% and 11.2%) and inter-day precision (16.5% and 15.6%) for TC and OTC in chicken samples. Moudgil et al. (2019b) carried out a study on accuracy and precision for TC and OTC. He reported that the recovery of accuracy was in the range 90.2%–108.1% for TC and was 99.2%–105.6% for OTC. The RSDs of the intra- and inter-assay precision for TC were 4.7% and 6.7–9.2%, while those for OTC were 6.2% and 9.6–16.2%, respectively.

Numerous investigations have been conducted to identify the presence of antibiotic residues in animal products in Asia, such as in Vietnam and China (Baghani et al., 2019), and the prevalence of antibiotic residues in chicken meat has been reported (Vandenberge et al., 2012; Sarker et al., 2018; Gompo et al., 2020; Maharjan et al., 2020; Rahman et al., 2021). In this study, an alarming number of samples were found to be positive for CIP (quinolone group), with 14.3% liver, 17.9% skin, and 3.6% muscle being above the MRLs in the raw stage, hence a potential health risk for consumption. The presence of CIP residues in the samples indicated that farmers in poultry production used this antibiotic as a therapeutic agent, which might have been excessively used or misused (Widiastuti et al., 2022). In developing countries, antibiotics are available as livestock medication without a prescription, and imported illegal products sometimes lack information about their use in the local language (Yamaguchi et al., 2015). In another study, Pereira et al. (2018) also mentioned that the detection of high frequencies of fluoroquinolone (including CIP) residues indicates the random use of these antibiotics due to the lack of proper monitoring by an authorized body. In the study of Panzenhagen et al. (2016), enrofloxacin (fluoroquinolone group) was found positive by HPLC in the muscles (23%), livers (17%), and kidneys (17%) from chickens. The mean concentrations of CIP found by Hasanen et al. (2016) in chicken thigh, breast, liver, and kidney were 92.11, 131.7, 211.1, and 316 µg/kg, while those in turkey thigh, breast, liver, and kidney were 83.2, 119.2, 205.7, and 291.9 µg/kg, respectively, which were higher than our findings. The results showed that approximately 10.71% muscle, 12.66% liver, and 15.38% skin samples exceeded the MRL (EU) among the positive samples of CIP, which were markedly high. In Malaysia, Marni et al. (2011) found that 10 out of 37 samples were contained ciprofloxacin (3.42–238.11 ng/g) and enrofloxacin in 33 samples (3.51–1,734.61 ng/g). Moreover, 45.7% of samples in a study in Turkey (Kaynak Onurdă Er. et al., 2013) and 59.2% of chicken meat samples in another study in Iran (Mashak et al., 2017) were found to be quinolone-positive. In Pakistan, Aslam et al. (2016) found that 58.3% (21) of meat samples had enrofloxacin residues above the MRL (100 ng/g). The findings of chicken samples containing CIP residues above the MRL of 100 ng/g in developing countries revealed the indiscriminate use of antibiotics, which is different from the picture in developed countries such as Spain, the UK, Portugal (Pereira et al., 2018), and Korea (Lee et al., 2018), where no samples exceeded the MRL of antibiotic residues. To reiterate, the differences in the results between the present study and other studies may be due to the multiple methods of administration of antibiotics in chicken, such as with feed and drinking water, intramuscularly, or intravenously (Abdullah et al., 2012; Ramatla et al., 2017). Furthermore, the detection rate of antibiotics may depend on the length of their use in chickens, slaughter, and sampling (Yamaguchi et al., 2015). Little change was found in the concentration of CIP in the positive samples after freezing, whereas a significant difference was observed in the study of O Aboul El-Nile (2006), who stated that temperatures below −18°C can have a positive effect on the reduction of CIP residues. In this study, in the boiling stage, the mean concentration of CIP was reduced from 74.49 to 50.72 µg/kg in the muscle, from 184.6 to 140.4 µg/kg in the liver, and from 79.75 to 49.15 µg/kg in the skin. In contrast, Hasanen et al. (2016) found that the CIP concentrations in both chicken and turkey did not change after boiling, grilling, and roasting, whereas these were notably reduced after microwave treatment and freezing for 1 month at −20°C. In support of this, Botsoglou and Fletouris (2001) also found that CIP has a high heat stability and is not easily destroyed at cooking temperature. In this study, the TC-positive samples in the raw stage were from 7.1% to 71.4%, with 7.1% of these found to be above the MRL; the OTC-positive samples were 3.6%–21.4%, but no sample was above the MRL. Bahmani et al. (2020) reported that TC residues were present in 54.3–389.1 µg/kg of the chicken samples, which was comparable to our data (40–353.1 µg/kg). According to Miranda et al. (2009), the TC and OTC residues in poultry meat ranged from 197 to 2,564 µg/kg and from 651 to 1,003 µg/kg, respectively, which were higher than our findings. In a study in Nepal, TCs and other antibiotics exceeded the MRLs in 6 out of 66 samples (Raut et al., 2017). This could be explained by marketing representatives promoting the use of these medications with easy affordability to poultry farmers, who then administered them without the assistance of trained veterinary officers and with lack of compliance to the specified withdrawal periods (Odundo et al., 2023). TCs can induce the overproduction of *Pseudomonas* and *Clostridium*, which change the normal intestinal flora (Hassan et al., 2020) and cause diarrhea, nausea, and even death. TCs consumed during pregnancy could remain in the structure of newly formed teeth (Muhammad et al., 2019). In Southern Italy, low-frequency OTC levels were reported in the muscle (3%) and liver (7%), showing a similar trend to that of the present study (Cammilleri et al., 2019). In poultry farming, OTC is used for the prevention of mouth infections and pneumonia, which can vary with the change of season depending on humidity (Aalipour et al., 2013). In addition, weather and seasonal variations are important factors that change the rate of a drug’s metabolism in chicken (Cerveny et al., 2021). Al-Ghamdi et al. (2000) found that the TC and OTC levels in beef and liver were lowered after boiling, and according to the observation of Gratacós-Cubarsí et al. (2007), boiling and microwave cooking lower the initial quantities of TC and OTC residues by 56% to 82%, respectively. Similarly, Abou-Raya et al. (2013) observed that the residual concentrations of TC and OTC were reduced by 25.8%–28.0% and 45.35%–67.05%, respectively, after boiling. All these have similarities to our findings.

The percent EDI-to-ADI ratio in 60-kg adults is used to measure the human health risk, whereas ADI is a standard criterion for assessing the safety of chemical contaminants in edible animal tissues. The present data for health risk assessment showed that the HI values for CIP, TC, and OTC from all chicken samples in the three stages were below 1, indicating low-risk exposure for human health. Oyedeji et al. (2019) analyzed the antibiotic residues from imported turkey-chicken products in Nigeria and found that the concentrations of 19 antibiotic residues were within the permissible limits. The HI for CIP in all the samples analyzed was less than 1 (HI < 1), indicating no risk for an adult population in Indonesia (Widiastuti et al., 2022). Pena et al. (2010) in Portugal and Pereira et al. (2018) revealed that the EDIs for enrofloxacin in chickens were 0.029 and 0.46 ng/kg body weight per day, respectively. The estimated daily exposure dose was found below 1 µg/kg bw per day with the presence of TCs, quinolones (QNLs), and sulfonamide antibiotics (SAs) in poultry meat in Shanghai (Wang et al., 2017). From 2012 to 2017, Kyriakides et al. (2020) studied antibiotic exposure from the consumption of pork meat in children and adolescents and found a low HI (below 0.056). These trends were also observed in our results. However, Vragovi´c et al. (2011) obtained significantly higher EDIs for streptomycin than for TCs in meat samples. Antibiotic exposure, even in low doses, develops antibiotic-resistant microorganisms, which could have long-term consequences on human health (Karbiwnyk et al., 2007; Gomes and Kuyucu, 2017; Kyuchukova, 2020). Dietary exposure to antibiotic residues could cause therapeutic failure, particularly in children (Jahantigh and Dizaji, 2015). Individuals who inadvertently consume antibiotic residues through edible chicken parts could suffer from allergies, from mutagenic, teratogenic, and anaphylactic shocks, and digestive disorders (Mund et al., 2016; Jammoul and El Darra, 2019). Arsène et al. (2022) also reported adverse health effects such as reproductive disorders, mutagenicity, alteration of the gut flora, and carcinogenicity, which can be caused by exposure to antibiotic-containing food. Cross-resistance could be produced in a new group of microorganisms from the antibiotic-resistant group due to structural alterations in the enzyme from the mutated gene (Sköld, 2001; Baran et al., 2011; Dimitrios et al., 2018).

## Conclusion

This study demonstrated two methods for the determination and validation of CIP, TC, and OTC. These methods were efficient in determining the CIP, TC, and OTC from different parts of poultry samples, which were confirmed by recovery studies. A great number of samples were found to be positive with antibiotics. The overall concentration of each antibiotic detected in broiler chicken was higher in raw samples than those in frozen and boiled samples. A few samples contained antibiotics in concentrations higher than their MRLs even after heat treatment, indicating a hazardous situation for human health. The results from the health risk assessment showed that low exposure to antibiotic residues may be a possible human health concern. In countries such as Bangladesh, where most of the poor and middle-class people are somehow dependent on broiler chicken as a cheaper source of protein and which has become a part of their consumption, it is important for authorities to pay attention to the use of veterinary drugs and to take necessary steps to avoid antibiotic resistance.

## Data availability statement

The original contributions presented in the study are included in the article/supplementary material. Further inquiries can be directed to the corresponding author.

## Ethics statement

The animal studies were approved by Ethical Review Committee (ERC), Mawlana Bhashani Science and Technology University, Tangail, Bangladesh. The studies were conducted in accordance with the local legislation and institutional requirements. Written informed consent was obtained from the owners for the participation of their animals in this study.

## Author contributions

SH: Data curation, Formal analysis, Investigation, Methodology, Software, Writing – original draft, Writing – review & editing. MJ: Data curation, Formal analysis, Investigation, Methodology, Software, Writing – original draft, Writing – review & editing. MK: Formal analysis, Investigation, Methodology, Validation, Visualization, Writing – original draft, Writing – review & editing. MR: Data curation, Formal analysis, Investigation, Methodology, Resources, Software, Visualization, Writing – original draft, Writing – review & editing. ME: Data curation, Software, Writing – review & editing. MA: Data curation, Resources, Software, Writing – review & editing. MZ: Project administration, Validation, Visualization, Writing – original draft, Writing – review & editing. MS: Data curation, Resources, Software, Writing – review & editing. LB: Conceptualization, Funding acquisition, Methodology, Project administration, Resources, Supervision, Validation, Visualization, Writing – original draft, Writing – review & editing.
